# A guide to best practices for Gene Ontology (GO) manual annotation

**DOI:** 10.1093/database/bat054

**Published:** 2013-07-09

**Authors:** Rama Balakrishnan, Midori A. Harris, Rachael Huntley, Kimberly Van Auken, J. Michael Cherry

**Affiliations:** ^1^Saccharomyces Genome Database, Department of Genetics, Stanford University, 300 Pasteur Drive, MC-5477 Stanford, CA 94305, USA, ^2^PomBase, Cambridge Systems Biology Centre, Department of Biochemistry, University of Cambridge, Sanger Building, 80 Tennis Court Road, Cambridge CB2 1GA, UK, ^3^UniProt, European Bioinformatics Institute, Wellcome Trust Genome Campus, Hinxton, Cambridgeshire CB10 1SD, UK and ^4^WormBase, Division of Biology, California Institute of Technology, 1200 E. California Boulevard, Pasadena, CA 91125, USA

## Abstract

The Gene Ontology Consortium (GOC) is a community-based bioinformatics project that classifies gene product function through the use of structured controlled vocabularies. A fundamental application of the Gene Ontology (GO) is in the creation of gene product annotations, evidence-based associations between GO definitions and experimental or sequence-based analysis. Currently, the GOC disseminates 126 million annotations covering >374 000 species including all the kingdoms of life. This number includes two classes of GO annotations: those created manually by experienced biocurators reviewing the literature or by examination of biological data (1.1 million annotations covering 2226 species) and those generated computationally via automated methods. As manual annotations are often used to propagate functional predictions between related proteins within and between genomes, it is critical to provide accurate consistent manual annotations. Toward this goal, we present here the conventions defined by the GOC for the creation of manual annotation. This guide represents the best practices for manual annotation as established by the GOC project over the past 12 years. We hope this guide will encourage research communities to annotate gene products of their interest to enhance the corpus of GO annotations available to all.

**Database URL:** http://www.geneontology.org

## Introduction

The Gene Ontology Consortium (GOC; http://www.geneontology.org) is a bioinformatics resource that serves as a comprehensive repository of functional information about gene products assembled through the use of domain-specific ontologies (1). The project is a collaborative effort working to describe how and where gene products act by creating evidence-supported gene-product annotations to structured comprehensive controlled vocabularies. The Gene Ontology (GO) is a controlled vocabulary composed of >38 000 precise defined phrases called GO terms that describe the molecular actions of gene products, the biological processes in which those actions occur and the cellular locations where they are present. First developed in 1998 (2), the GOC project has grown to become an integrated resource providing functional information for a wide variety of species. As of January 2013, there are >126 million annotations to >19 million gene products from species throughout the tree of life. Of these there are 1.1 million manually curated annotations, from published experimental results, to 234 000 gene products. As the GOC develops the standard language to describe function, it also defines standards for using these ontologies in the creation of annotations. This article elaborates on the methods and conventions adopted by the GOC curation teams for constructing annotations and serves as a guide to new or potential annotators, and the biological community at large, for understanding the requirements necessary to create and maintain the highest quality GO annotations.

## Overview of GO annotations

**Figure 1.**
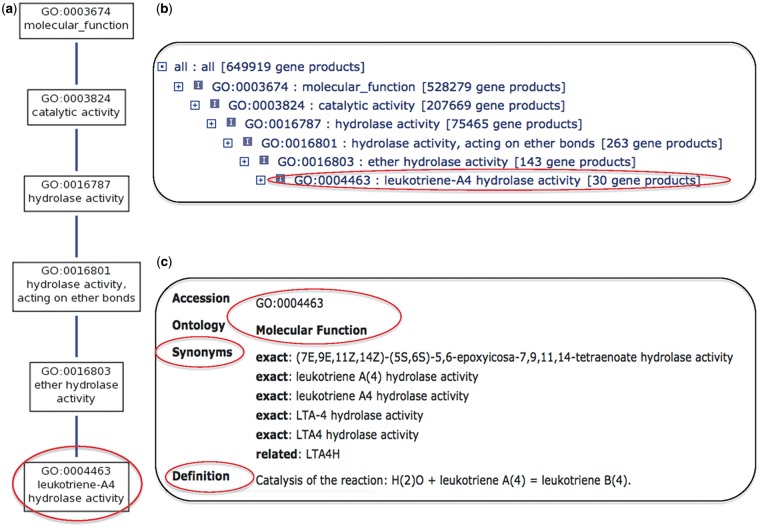
GO Term ‘leukotriene-A4 hydrolase activity’ [GO:0004463], one of the terms mentioned in the main text of the article, as seen in AmiGO (16, http://amigo.geneontology.org). (**a**) Graphical view of the ontology structure showing the most granular term ‘leukotriene-A4 hydrolase activity’ [GO:0004463] at the bottom (highlighted in red), and all its parent terms leading up to the root node (‘molecular_function’ [GO:0003674]) at the top. Each box representing a GO term includes the GO identifier, and the blue line connecting the terms represent the ontological relationship ‘*is_a*’ (implying that a child term is a subtype of the parent term). (**b**) Alternate text display for viewing the ontology structure. ‘leukotriene-A4 hydrolase activity’ [GO:0004463] is highlighted in red. Each child term is indented from its parent to indicate the depth of the tree. Apart from the GOID and GO term, each row includes other pieces of information that are important to understand the ontology and the annotations to each term. Starting from the left end of the row, the + sign indicates that there are child terms for that node and clicking on the + sign opens the browser to display the child terms. Next the small icon ‘i’ indicates the term is related to its parent by an *is–a* relationship (explained above). At the right end of the row in brackets is the total number of gene products annotated to that term and all its child terms. (**c**) Term information relevant to making an annotation is highlighted in red, which includes the GOID, Aspect of the ontology (Molecular Function), Synonyms and Definition of the term.

The goal of the GOC is the unification of biology by creating a nomenclature used for describing the functional characteristics of any gene product, protein or RNA, from any organism. There are two parts to a GO annotation: first, the association asserted between a gene product and a GO definition; and second, the source (e.g. published article) and evidence used as the authority to make the assertion. The GO is a set of highly structured directed acyclic graphs (DAGs); its structure and content have been extensively described elsewhere (2, 3). Here, we limit our presentation to the GO term name (the phrase that is typically used when discussing individual components of the ontologies, often shortened to ‘GO term’), the GO definition, the text string that explains the precise meaning of the GO term and a numerical identifier called the GOID (examples used in this guide are shown in Table 1). In addition, each term can have multiple ontological relationships to broader (parent) and more specific (child) terms (Figure 1 illustrates how terms and relationships are represented in GO).

**Table 1.** This table lists a small subset of the GO terms used as examples in the main text of the article

GOID	Aspect	GO term name	Definition
GO:0003674	Molecular function	Molecular_function	Elemental activities, such as catalysis or binding, describing the actions of a gene product at the molecular level. A given gene product may exhibit one or more molecular functions.
GO:0008150	Biological process	Biological_process	Any process specifically pertinent to the functioning of integrated living units: cells, tissues, organs and organisms. A process is a collection of molecular events with a defined beginning and end.
GO:0005575	Cellular component	Cellular_component	The part of a cell or its extracellular environment in which a gene product is located. A gene product may be located in one or more parts of a cell and its location may be as specific as a particular macromolecular complex, that is, a stable, persistent association of macromolecules that function together.
GO:0004672	Molecular function	Protein kinase activity	Catalysis of the phosphorylation of an amino acid residue in a protein, usually according to the reaction: a protein + ATP = a phosphoprotein + ADP.
GO:0022858	Molecular function	Alanine transmembrane transporter activity	Catalysis of the transfer of alanine from one side of a membrane to the other. Alanine is 2-aminopropanoic acid.
GO:0003872	Molecular function	6-phosphofructokinase activity	Catalysis of the reaction: ATP + D-fructose-6-phosphate = ADP + D-fructose 1,6-bisphosphate.
GO:0000981	Molecular function	Sequence-specific DNA binding RNA polymerase II transcription factor activity	Interacting selectively and noncovalently with a specific DNA sequence in order to modulate transcription by RNA polymerase II. The transcription factor may or may not also interact selectively with a protein or macromolecular complex.
GO:0000988	Molecular function	Protein binding transcription factor activity	Interacting selectively and noncovalently with any protein or protein complex (a complex of two or more proteins that may include other nonprotein molecules), in order to modulate transcription. A protein binding transcription factor may or may not also interact with the template nucleic acid (either DNA or RNA) as well.
GO:0043565	Molecular function	Sequence-specific DNA binding	Interacting selectively and noncovalently with DNA of a specific nucleotide composition, e.g. GC-rich DNA binding, or with a specific sequence motif or type of DNA e.g. promotor binding or rDNA binding.
GO:0004674	Molecular function	Protein serine/threonine kinase activity	Catalysis of the reactions: ATP + protein serine = ADP + protein serine phosphate, and ATP + protein threonine = ADP + protein threonine phosphate.
GO:0004712	Molecular function	Protein serine/threonine/tyrosine kinase activity	Catalysis of the reactions: ATP + a protein serine = ADP + protein serine phosphate; ATP + a protein threonine = ADP + protein threonine phosphate; and ATP + a protein tyrosine = ADP + protein tyrosine phosphate.
GO:0005515	Molecular function	Protein binding	Interacting selectively and noncovalently with any protein or protein complex (a complex of two or more proteins that may include other nonprotein molecules).
GO:0042393	Molecular function	Histone binding	Interacting selectively and noncovalently with a histone, any of a group of water-soluble proteins found in association with the DNA of plant and animal chromosomes. They are involved in the condensation and coiling of chromosomes during cell division and have also been implicated in nonspecific suppression of gene activity.
GO: 0016887	Molecular function	ATPase activity	Catalysis of the reaction: ATP + H2O = ADP + phosphate + 2 H+. May or may not be coupled to another reaction.
GO:0008121	Molecular function	Ubiquinol-cytochrome-c reductase activity	Catalysis of the transfer of a solute or solutes from one side of a membrane to the other according to the reaction: CoQH2 + 2 ferricytochrome c = CoQ + 2 ferrocytochrome c + 2 H+.
GO:0004463	Molecular function	Leukotriene-A4 hydrolase activity	Catalysis of the reaction: H(2)O + leukotriene A(4) = leukotriene B(4).
GO:0008152	Biological process	Metabolic process	The chemical reactions and pathways, including anabolism and catabolism, by which living organisms transform chemical substances. Metabolic processes typically transform small molecules, but also include macromolecular processes such as DNA repair and replication, and protein synthesis and degradation.
GO:0023052	Biological process	Signaling	The entirety of a process in which information is transmitted within a biological system. This process begins with an active signal and ends when a cellular response has been triggered.
GO:0016265	Biological process	Death	A permanent cessation of all vital functions: the end of life; can be applied to a whole organism or to a part of an organism.
GO:0008219	Biological process	Cell death	Any biological process that results in permanent cessation of all vital functions of a cell. A cell should be considered dead when any one of the following molecular or morphological criteria is met: (i) the cell has lost the integrity of its plasma membrane; (ii) the cell, including its nucleus, has undergone complete fragmentation into discrete bodies (frequently referred to as ‘apoptotic bodies’); and/or (iii) its corpse (or its fragments) have been engulfed by an adjacent cell *in vivo*.
GO:0006915	Biological process	Apoptotic process	A programmed cell death process which begins when a cell receives an internal (e.g. DNA damage) or external signal (e.g. an extracellular death ligand), and proceeds through a series of biochemical events (signaling pathways) which typically lead to rounding-up of the cell, retraction of pseudopodes, reduction of cellular volume (pyknosis), chromatin condensation, nuclear fragmentation (karyorrhexis), plasma membrane blebbing and fragmentation of the cell into apoptotic bodies. The process ends when the cell has died. The process is divided into a signaling pathway phase and into an execution phase, which is triggered by the former.
GO:0030263	Biological process	Apoptotic chromosome condensation	The compaction of chromatin during apoptosis.
GO:0032329	Biological process	Serine transport	The directed movement of l-serine, 2-amino-3-hydroxypropanoic acid, into, out of or within a cell, or between cells, by means of some agent such as a transporter or pore.
GO:0015826	Biological process	Threonine transport	The directed movement of threonine, (2R*,3S*)-2-amino-3-hydroxybutanoic acid, into, out of or within a cell, or between cells, by means of some agent such as a transporter or pore.
GO:0032328	Biological process	Alanine transport	The directed movement of alanine, 2-aminopropanoic acid, into, out of or within a cell, or between cells, by means of some agent such as a transporter or pore.
GO:00034605	Biological process	Cellular response to heat	Any process that results in a change in state or activity of a cell (in terms of movement, secretion, enzyme production, gene expression, etc.) as a result of a heat stimulus, a temperature stimulus above the optimal temperature for that organism.
GO:0071470	Biological process	Cellular response to osmotic stress	Any process that results in a change in state or activity of a cell (in terms of movement, secretion, enzyme production, gene expression, etc.) as a result of a stimulus indicating an increase or decrease in the concentration of solutes outside the organism or cell.
GO:0034599	Biological process	Cellular response to oxidative stress	Any process that results in a change in state or activity of a cell (in terms of movement, secretion, enzyme production, gene expression, etc.) as a result of oxidative stress, a state often resulting from exposure to high levels of reactive oxygen species, e.g. superoxide anions, hydrogen peroxide (H_2_O_2_), and hydroxyl radicals.
GO:0033554	Biological process	Cellular response to stress	Any process that results in a change in state or activity of a cell (in terms of movement, secretion, enzyme production, gene expression, etc.) as a result of a stimulus indicating the organism is under stress. The stress is usually, but not necessarily, exogenous (e.g. temperature, humidity, ionizing radiation).
GO:0006351	Biological process	Transcription, DNA dependent	The cellular synthesis of RNA on a template of DNA.
GO:0006357	Biological process	Regulation of transcription from RNA polymerase II promoter	Any process that modulates the frequency, rate or extent of transcription from an RNA polymerase II promoter.
GO:0007067	Biological process	Mitosis	A cell cycle process comprising the steps by which the nucleus of a eukaryotic cell divides; the process involves condensation of chromosomal DNA into a highly compacted form. Canonically, mitosis produces two daughter nuclei whose chromosome complement is identical to that of the mother cell.
GO:0000084	Biological process	S phase of mitotic cell cycle	S phase occurring as part of the mitotic cell cycle. S phase is the part of the cell cycle during which DNA synthesis takes place. A mitotic cell cycle is one which canonically comprises four successive phases called G1, S, G2 and M and includes replication of the genome and the subsequent segregation of chromosomes into daughter cells.
GO:0031028	Biological process	Septation initiation signaling cascade	The series of molecular signals, mediated by the small GTPase Ras, that results in the initiation of contraction of the contractile ring, at the beginning of cytokinesis and cell division by septum formation. The pathway coordinates chromosome segregation with mitotic exit and cytokinesis.
GO:0051321	Biological process	Meiotic cell cycle	Progression through the phases of the meiotic cell cycle, in which canonically a cell replicates to produce four offspring with half the chromosomal content of the progenitor cell.
GO:0005634	Cellular component	Nucleus	A membrane-bounded organelle of eukaryotic cells in which chromosomes are housed and replicated. In most cells, the nucleus contains all of the cell’s chromosomes except the organellar chromosomes, and is the site of RNA synthesis and processing. In some species, or in specialized cell types, RNA metabolism or DNA replication may be absent.
GO:0005681	Cellular component	Splicesosomal complex	Any of a series of ribonucleoprotein complexes that contain RNA and small nuclear ribonucleoproteins (snRNPs), and are formed sequentially during the splicing of a messenger RNA primary transcript to excise an intron.
GO:0044428,	Cellular component	Nuclear part	Any constituent part of the nucleus, a membrane-bounded organelle of eukaryotic cells in which chromosomes are housed and replicated.
GO:0005826	Cellular component	Actomyosin contractile ring	A cytoskeletal structure composed of actin filaments and myosin that forms beneath the plasma membrane of many cells, including animal cells and yeast cells, in a plane perpendicular to the axis of the spindle, i.e. the cell division plane. Ring contraction is associated with centripetal growth of the membrane that divides the cytoplasm of the two daughter cells. In animal cells, the contractile ring is located inside the plasma membrane at the location of the cleavage furrow. In budding fungal cells, e.g. mitotic *S. cerevisiae* cells, the contractile ring forms beneath the plasma membrane at the mother-bud neck before mitosis.
GO:0005750	Cellular component	Mitochondrial respiratory chain complex III	A protein complex located in the mitochondrial inner membrane that forms part of the mitochondrial respiratory chain. Contains about 10 polypeptide subunits including four redox centers: cytochrome b/b6, cytochrome c1 and an 2Fe-2S cluster. Catalyzes the oxidation of ubiquinol by oxidized cytochrome c1.

The GO Term is a short phrase that is typically used to represent the individual components of the ontologies, while the definitions provide the precise meaning of the GO terms. As emphasized in the text, it is important to create annotations to the definition and not to the GO Term. Curators should explore the ontologies using AmiGO (17, http://amigo.geneontology.org) or QuickGO (18, http://www.ebi.ac.uk/QuickGO/) to identify appropriate terms for annotation. More information on the structure of the Gene Ontology and how it is developed is available online from http://www.geneontology.org.

Although annotations are typically viewed as connections between a gene product and a GO term, it is important to stress that the GO term name is a surrogate for the definition, and that the biological concept described by the definition is really the core assertion being made by an annotation. This is a subtle yet important point central to understanding the power of the GO, one that is not always appreciated by both annotators and consumers of GO annotations. As with a spoken language, the understanding of its usage is based on shared definitions of the phrases and definitions of the terms. Thus, annotating to the definition is required to alleviate confusion if the names of biological concepts or terminology used in the published literature are ambiguous.

The source of the information used to make an annotation includes both a specific reference, usually a published scientific article represented by a PubMed identifier (PMID), that describes the result of an experimental or computational analysis on which the association was based, and an evidence code (Table 2) that reflects the type of experimental assay or analysis that supports the association. Annotations can be asserted manually from the literature by biocurators or computationally by automated methods. This article will focus on standards defined by the GOC for manual curation. Computational annotation methods and their guidelines have been reported elsewhere (4).

**Table 2.** Evidence code categories, evidence code names and definitions

Category	Evidence code	Types of evidence	Supporting data
Experimental	Inferred from Experiment (EXP)	Any experimental assay	
	Inferred from Direct Assay (IDA)	(i) Enzyme assays	
		(ii) *In vitro* reconstitution (e.g. transcription)	
		(iii) Immunofluorescence (for cellular component)	
		(iv) Cell fractionation (for cellular component)	
		(v) Physical interaction/binding assay (sometimes appropriate for cellular component or molecular function)	
	Inferred from Physical Interaction (IPI)	(i) Two-hybrid interactions	With column should be filled with Identifier of the interacting protein
		(ii) Co-purification	
		(iii) Co-immunoprecipitation	
		(iv) Ion/protein binding experiments	
	Inferred from Mutant Phenotype (IMP)	(i) Mutations, natural or introduced, that result in partial or complete impairment or alteration of the function of that gene	
		(ii) Polymorphism or allelic variation (including where no allele is designated wild-type or mutant)	
		(iii) Any procedure that disturbs the expression or function of the gene, including RNAi, anti-sense RNAs, antibody depletion, or the use of any molecule or experimental condition that may disturb or affect the normal functioning of the gene, including: inhibitors, blockers, modifiers, any type of antagonists, temperature jumps, changes in pH or ionic strength	
		(iv) Overexpression or ectopic expression of wild-type or mutant gene	
	Inferred from Genetic Interaction (IGI)	(i) ‘Traditional’ genetic interactions such as suppressors, synthetic lethals, etc.	With column should be filled with identifier of the interacting gene
		(ii) Functional complementation	
		(iii) Rescue experiments	
		(iv) Inference about one gene drawn from the phenotype of a mutation in a different gene	
	Inferred from Expression Pattern (IEP)	(i) Transcript levels or timing (e.g. Northerns, microarray data)	
		(ii) Protein levels (e.g. Western blots)	
Computational Analysis	Inferred by Sequence Similarity (ISS)	Sequence, structural similarity-based analysis	With column should be filled with identifier of the similar gene/protein
	Inferred by Sequence Alignment (ISA)	Pairwise or multiple alignment	With column should be filled with identifier of the similar gene/protein
	Inferred by Sequence Orthology (ISO)	Assertion of orthology between the gene product and a gene product in another organism	With column should be filled with identifier of the similar gene/protein
	Inferred from Sequence Model (ISM)	Prediction methods for noncoding RNA genes such as tRNASCAN-SE, Snoscan, and Rfam	
	Inferred from genomic context (IGC)	Information about the genomic context of a gene product forms part of the evidence for a particular annotation.	
		(i) operon structure	
		(ii) syntenic regions	
		(iii) pathway analysis	
		(iv) genome scale analysis of processes	
	Inferred from Key Residues (IKR)	Sequence analysis, where lack of key sequence residues is used to make a negative (NOT) annotation	Should include NOT qualifier and With column is required if analysis is carried out by a curator and GO_reference:0000047
Author	Traceable Author Statement (TAS)	Original evidence is referenced in the article and therefore can be traced to another source	
	Non-traceable Author Statement (NAS)	Statements in articles that cannot be traced to another article or experiment	
Curatorial	No Data (ND)	Used for annotations when information about the molecular function, biological process, or cellular component of the gene or gene product being annotated is not available	Should be used only with root nodes
	Inferred by Curator (IC)	Annotation reasonably inferred by a curator from other GO annotations, for which direct evidence is available	GO ID should be filled in the from column

The evidence codes are used to represent the method or type of results used to define the annotation. Annotators should use this table as a quick reference guide and consult the detailed documentation available online (http://www.geneontology.org/GO.evidence.shtml) for specific details (including the do’s and don’ts) on how the method or results are matched to each evidence code.

## Annotation format

GO annotations are recorded and supplied in a standard tab-delimited file format called the Gene Associations File (GAF, http://www.geneontology.org/GO.format.annotation.shtml). For each annotation, the GAF format contains both required and optional fields, some of which will be discussed below. The required fields are—the identifier of the gene product being annotated, the GOID of the GO term associated with the gene product, an evidence code and the reference (either a published article or a GOC-specific internal reference) supporting the use of the GOID, the aspect of the ontology (Molecular Function, Biological Process, Cellular Component), the curation project that created the annotation, the object type that is being annotated (see below), the NCBI taxonomy database identifier for the species of the gene product and the date the annotation was created or modified. A sample annotation is shown in Table 3.

**Table 3.** A sample annotation in the GAF 2.0 format

Column	Content	Required?	Example
1	DB	Required	MGI
2	DB Object ID	Required	MGI:1350922
3	DB Object Symbol	Required	Cadps
4	Qualifier	Optional	NOT
5	GO ID	Required	GO:0006887
6	DB:Reference (|DB:Reference)	Required	MGI:MGI:3583730|PMID:15820695
7	Evidence Code	Required	IMP
8	With (or) From	Optional	MGI:MGI:3583931
9	Aspect	Required	P
10	DB Object Name	Optional	Ca2+-dependent secretion activator
11	DB Object Synonym (|Synonym)	Optional	CAPS1
12	DB Object Type	Required	Protein
13	Taxon(|taxon)	Required	Taxon:10090
14	Date	Required	20060202
15	Assigned By	Required	MGI
16	Annotation Extension	Optional	Occurs_in(CL:0000001)|occurs_in(CL:0000336)
17	Gene Product Form ID	Optional	UniProtKB:Q80TJ1

This table provides an example of an annotation from the Mouse Genome Informatics group (from February 2013). The Cadps protein (MGI identifier MGI:1350922) was annotated by the MGI project to ‘exocytosis’ [GO:0006887], a term in the Biological Process ontology indicated by ‘P’ in column 9. This annotation used the ‘NOT’ qualifier indicating the authors of PMID:15820695 (5) showed that this protein is ‘NOT’ involved in ‘exocytosis’. The non-PMID reference number, MGI:MGI:3583730, is MGI’s internal identifier for the same reference. The curators arrived at this annotation based on the phenotype of the Cadps mutant, which is indicated with the IMP evidence code. The identifier of the allele (MGI:MGI:3583931) used in the experiment is captured in column 8 (WITH/FORM). In addition, the annotation extension field (column 16) indicates the cell types where this protein (CL:0000001, primary cell culture or CL:0000336, adrenal medulla chromaffin cell) was NOT found to be involved in this process (exocytosis). Finally, the last column represents the UniProtKB identifier for the isoform of the mouse Cadps protein that was studied.

## Manual curation

Within the GOC, manual annotations are made by experienced biocurators from a variety of annotation projects including, but not limited to, the *Saccharomyces* Genome Database [SGD, (6)], Mouse Genome Informatics [MGI, (7)], WormBase (8), PomBase (9), FlyBase (10), ZFIN (11) and UniProt (12). Manual curation typically encompasses two approaches. The first involves reading relevant publications, identifying the gene product(s) of interest, and ascribing the reported experimental results to a GO definition using an appropriate evidence code (Table 2). The second involves inferring a gene’s role by manual examination of its nucleic acid or protein sequence motifs, structure or phylogenetic relationships. For consistent interpretation of experimental results and sequence analysis, the GOC has established annotation guidelines that are elaborated below. GOC member projects (http://www.geneontology.org/GO.consortiumlist.shtml) with assistance from other groups engaged in advancing the representation of biological function so that it can be presented in a straightforward but precisely defined form have developed these guidelines. Over time these guidelines have evolved into required standards for all manual annotations and have been incorporated into validation tools used by the GOC to maintain their quality and uniformity.

## Gene product: Object of annotation

The annotation object or molecular entity are those defined by the Sequence Ontology [(13), http://www.sequenceontology.org] and includes complex, gene, gene_product, miRNA, ncRNA, protein, protein_complex, protein_structure, RNA, rRNA, snoRNA, snRNA, transcript, tRNA and polypeptide. While annotations are typically created for chromosomal features, such as a gene for its protein or ncRNA product, other types of objects can be annotated including groups of gene products that make a complex. The annotation object can be associated to a GO term from one or more of the three aspects of the GO (Molecular Function, Biological Process and Cellular Component). A gene product is the most common object of annotation, and all such objects require a stable identifier such as those specified by sequence databases maintained by European Bioinformatic Institute (EBI) and National Center for Biotechnology Information (NCBI). Model Organism Databases (MODs) also maintain unique identifiers that often represent specific types of molecular entities such as RNA transcripts that often do not have an identifier from one of the archival repositories.

## Approaching an article for curation

When experimental data on a gene product has been published, the following guidelines can be used to identify the relevant or annotatable pieces of information that may generate GO annotation for that gene product.

- Identification of relevant articles describing a gene product’s function is the essential starting point for making annotations. While PubMed is a typical starting point for finding relevant articles, research in the area of Natural Language Processing (NLP) provides additional methods that can aid in the search for curatable articles. More on NLP methods used for biocuration can be found in the reports from the Biocreative workshops (14). Once an article has been identified, biocurators must properly specify the objects of annotation including confirmation of the correct taxa. These details are often found in the Methods section of the article, but unambiguously determining species for annotation can be problematic, particularly in vertebrate systems where orthologous gene names are shared among taxa. Further, when multiple model organism systems are being used simultaneously, the taxa of the genes being investigated is not always specifically designated. For example, Lin and Isaacson (15) studied axonal growth regulation by netrin and slit proteins using both mouse and rat cells. Two of the plasmids containing slit coding sequences were acknowledged as gifts and no reference to the species of origin was provided. In this case, to determine the species the sequences represent, the authors had to be contacted to confirm that the sequences actually originated from human, neither mouse or rat.
- The Introduction section of the article will often present previous knowledge about the gene product’s function. If citations to original works are included then the article can be used as a source of the information and annotated using the evidence code (see below) *Traceable Author Statement* (TAS). The use of TAS evidence has decreased over time, as it is best practice to go to the original article to capture the annotation directly from experimental results. This allows for clear attribution of an annotation to the original experimental details. Thus, GOC strongly discourages the continued use of TAS and recommends replacing existing TAS annotations with those to the published experimental results.
- Annotations derived from experimental data are most often found in the Methods and Results sections or in the figure legends of articles. A biocurator can efficiently receive an overview of the biological context of the article from the Introduction section and then, using the experimental data in the Results section, create annotations with appropriate supporting experimental evidence.
- Authors often speculate on the role of the gene product in the Discussion section based on the experimental results they present. The authors may propose a hypothesis that combines previous knowledge, new findings from the current study and new ideas that have not yet been experimentally verified. This information is not suitable for an annotation assertion and if used to create an annotation can be detrimental, as these hypotheses have not been validated.

## Manual curation using sequence similarity data

Manual curation by biocurators includes the *in-silico* analysis of chromosomal features to infer a gene product’s role and location. GO terms can be assigned to gene products on the basis of sequence similarity using the evidence code 'Inferred from Sequence or structural Similarity' (ISS) with a custom reference, GO_Reference (GO_REF:0000024), as described in the next section. Potential homologs are initially identified using sequence similarity search programs such as BLAST. The significance of the sequence similarity is then verified manually using a combination of sequence resources and analysis tools, including phylogenetic and comparative genomics databases such as Ensembl Compara (16), INPARANOID (17) and OrthoMCL (18). In all cases, biocurators validate each alignment to assess whether similarity is appropriate to infer the gene product’s function. While there is no universal definition for the minimum requirements for similarity results, the significance of a match is judged on a case-by-case basis by the biocurator’s expertise. Although the similarity criteria required to make these annotations are defined by the annotating group, the GOC has established several rules for making these assignments. They are as follows:

- Mandatory inclusion of a stable database identifier that identifies the similar gene/gene product in the ‘WITH/FROM’ field (column 8 in Table 3)
- The similar gene must be experimentally characterized; to avoid circular inferences, the GO term should only be assigned if the similar gene/gene product is, or can be annotated, with the same term (or a more specific child term) using an experimental evidence code (e.g. Inferred from Direct assay, IDA; Inferred from Mutant Phenotype, IMP; Inferred from Genetic Interaction, IGI, Inferred from Physical Interaction, IPI; Inferred from Expression Pattern, IEP). Annotations made with the NOT qualifier should not be transferred.

Sequence characteristics can be used to infer GO annotations for all three aspects of the ontology. However, care should be taken when transferring biological process annotations, as cellular processes and metabolic processes, for example, may be more readily inferred from sequence similarity than developmental processes which may be species- or clade-specific

## Use of GO reference

As mentioned above, manual curation does not always require a published reference to indicate the source of evidence. Annotations can be inferred by biocurators by analysis of the gene sequence or by combining direct experimental evidence from multiple sources. In these situations, the citation is to a custom reference. These so-called *GO references* describe the methods and procedures used in creating such annotations. For example, GO_REF:0000024 (http://www.geneontology.org/cgi-bin/references.cgi#GO_REF:0000024), titled ‘Manual transfer of experimentally verified manual GO annotation data to orthologs by curator judgment of sequence similarity’, was created to describe the transfer of manual annotations using curator judgment to annotations associated with the ISS code. A second example is GO_REF:0000036 (http://www.geneontology.org/cgi-bin/references.cgi#GO_REF:0000036), ‘Manual annotations that require more than one source of functional data to support the assignment of the associated GO term.’ This GO reference is used with the *Inferred by Curator* (IC) evidence code, described below. GO references are created and published on the GOC Web site (http://www.geneontology.org/cgi-bin/references.cgi) only once the biocurators agree on the content of the abstract and its usage.

## How to define an annotation?

Once literature relevant to a gene product has been identified, the following guidelines can be used to decide which GO term(s) and evidence code(s) should be associated. Individual articles may not provide results that support annotations for all three aspects of the ontology; thus, annotations to the different aspects will generally need to come from different articles. Also it is common, from a single article, to identify multiple annotations identified for one aspect and to annotate to different levels of granularity in the same branch of the ontology. The granularity of the GO term selected depends heavily on the type of experiments being reported as well as the ability of the biocurator to understand the limitations of that experimental method. MacCullen (19) interviewed biocurators from the GOC in an effort to correlate the curator’s education, work experience and research experience to measured variability in annotation. After observing there was significant variability in a test set of annotations, he explored possible causes. MacCullen reported no correlation between the amount of variation and any specific characteristic of the biocurator’s education or experience and suggested that biocurators should continually work to coordinate annotation methods with the goal of minimizing variation. The solution used by the Consortium’s member projects is to have continuing education and discussions between biocurators to reduce variability that arises from inconsistent use of the rules and misunderstanding of the ontology terms. Also to further address the variability in the interpretation by biocurators, the GOC holds regular controlled annotation exercises to define standards and maintain consistent procedures. These exercises are conducted within and across most projects where biocurators annotate the same article or a small set of articles and then compare their annotations. A discussion follows where the GOC comes to a consensus about the most appropriate annotations for that article and in the process educates its staff.

## Choosing the right GO term

As emphasized above, ontology terms should be chosen based not on the term name, but on the definition of the term. Ontology terms can be explored using AmiGO (20), http://amigo.geneontology.org, or QuickGO (21), http://www.ebi.ac.uk/QuickGO/. Often it is hard to find the appropriate GO term using the description or phrases from the literature because GO terms can be more descriptive and they reflect the actual function or process rather than a gene product name or family name. Therefore, to assist in searching, and to accurately reflect the language of biology, many ontology terms are associated with synonyms, which are typically the terminology or language used in the literature. For example, the phrase ‘transcription repressor’ is loosely used in the literature to refer to any transcription repressing role. This concept is represented in the GO as ‘negative regulation of transcription, DNA-dependent’ [GO:0045892], and the phrase transcription repressor is a synonym of this term. Development of the ontologies (i.e., adding new terms, refining definitions) is an active process and if an appropriate GO term that is suitable to describe a gene product is not available, biocurators are encouraged to request that a new term be added to the ontology. The GOC has setup several ways to handle new term requests and to evaluate existing terms. The easiest way is to contact the GO helpdesk (go-helpdesk@geneontology.org or http://www.geneontology.org/GO.contacts.shtml) providing as much detail as possible.

## What if nothing is known about the gene product?

Typically after an organism’s genome sequence is determined, structural annotation is performed using computational methods to make gene model predictions. Some of the resulting predicted genes will have been previously characterized and as a result will have literature-associated evidence or sequence based relationships to other well-defined genes. For other predicted genes neither experimental nor sequence based functions will be available. This represents sets of similar proteins that have yet to be characterized and proteins without similarity to any previously characterized sequence. Thus no literature is available on which to base an annotation. In cases such as this where nothing can be gleaned from the literature, it is correct to associate the gene product to the most general terms in the three ontologies, ‘molecular_function’ (GO:0003674), ‘biological_process’ (GO:0008150) and ‘cellular_component’ (GO:0005575) (called the root nodes, see Table 1) with the evidence code *No Data* (ND). It should be noted that annotating to the root node specifically states that an extensive search of the literature was conducted and no experimental results were found to indicate the function of this gene product. Since a biocurator infers that nothing has been published about the gene product, a custom reference (not a published article) that documents this curatorial procedure (the ‘GO reference’ GO_REF:0000015) should be included in a ND annotation. These ND annotations are used by projects such as SGD that have hunted through the published literature for reported functions of all gene products in the budding yeast. In this way the users can trust that a literature search did indeed occur. The use of ND is important because the absence of an annotation could mean that a function has been reported but no GO annotation has been captured or that there is no evidence available. Annotation projects should routinely explore any newly published works describing genes in their area of interest to determine if any new experimental results are available. Once new annotations have been defined, existing ND annotations for that gene product should be removed.

It is especially important that biocurators make sure the results presented in the article fit all parts of the term definitions; biocurators should not rely only on the term name. In the following, we present guidelines for commonly encountered curation issues observed for the individual ontologies.

## Molecular function

**Figure 2.**
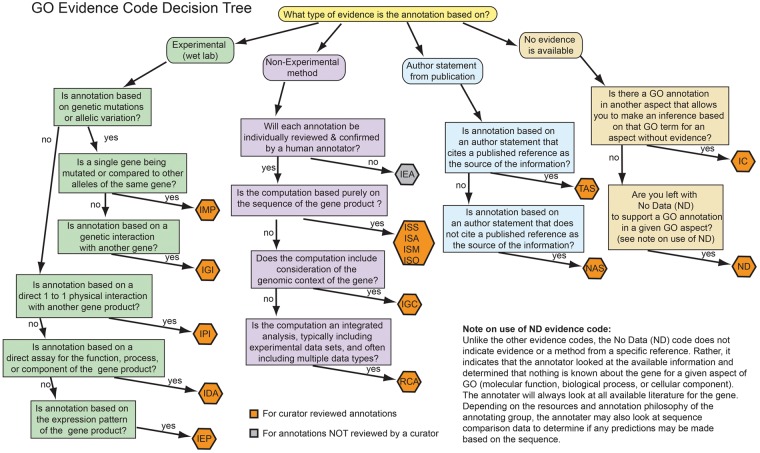
GO Evidence code decision tree describing the process of choosing an evidence code. This flow chart is meant to orient the biocurator on the different categories of evidence codes and does not include the complete definitions of the evidence codes (Table 2). This chart will aid the biocurator to evaluate the reported method or results and map them to an appropriate evidence code; the biocurator should consult the detailed evidence code documentation available online from http://www.geneontology.org/GO.evidence.shtml.

Molecular Function describes activities, such as catalytic, binding or transporter activities, at the molecular level (e.g. ‘protein kinase activity’ [GO:0004672], ‘6-phosphofructokinase activity’ [GO:0003872], ‘transcription factor binding’ [GO:0008134], ‘alanine transmembrane transporter activity’ [GO:0022858], see Table 1 for GOIDs and definitions for these GO terms). GO molecular function terms describe activities rather than the entities (complexes, gene products or molecules) that perform the actions. Typically direct assays such as enzyme kinetics measurements or binding studies can be used to infer molecular function annotations. In addition sequence comparison methods are often used to predict the molecular function of a gene product because functions are often associated with conserved protein domains (see Figure 2 to compare evidence from experimental and nonexperimental results).

- Deciding between a Molecular Function and a Biological Process term takes practice. The key question to ask when selecting a Molecular Function term is whether the experimental results show ‘how’ the gene product accomplishes its role. For example if the result simply shows that a mutant version of a gene product affects transcription, by itself that doesn’t show that the gene product is a transcription factor. If instead the study shows that transcription is modulated when the gene product binds to DNA or protein, then an appropriate Molecular Function term (‘sequence-specific DNA binding RNA polymerase II transcription factor activity’ [GO:0000981] or one of the child terms of ‘protein binding transcription factor activity’ [GO:0000988]) would be correct. In contrast, data from a mutant phenotype experiment could be used to make a Biological Process annotation to the term, ‘transcription, DNA-dependent’ [GO:0006351] or to one of its child terms (see Table 1 for GOIDs and definitions).
- Only GO terms that can be supported by the experimental results should be selected, based on the GO term definitions. For example, if the Introduction of an article states that a gene product is a transcription factor but only provides experimental results showing DNA binding, then this article is not appropriate for an experimentally based annotation to ‘sequence-specific DNA binding RNA polymerase II transcription factor activity’ [GO:0000988]. The appropriate term would be ‘sequence-specific DNA binding’ [GO:0043565] (see Table 1) or a more specific DNA binding term. In another situation, if the authors show via sequence comparison methods that a protein is a serine/threonine/tyrosine kinase, but only show experimental evidence for phosphorylation of serine and threonine, the biocurator must only annotate to ‘protein serine/threonine kinase activity’ [GO:0004674] using an experimental evidence code (example Inferred by Mutant Phenotype or Inferred by Direct Assay, see Figure 2). The biocurator could add an annotation to the protein serine/threonine/tyrosine kinase activity with ISS evidence code, see below. These annotations thus indicate what was experimentally shown in an article and what was predicted from sequence comparison.
- The Molecular Function ontology also contains terms that describe protein–protein interactions. However, annotating to such terms, e.g. ‘protein binding’ [GO:0005515], is done with careful consideration, as most proteins bind other proteins at one time or another. A rule of thumb is to determine whether the gene product being annotated is accomplishing a biological purpose by binding to another protein: if so, protein binding could be one of its functions. If more specific information on the type of protein being bound is available then the annotation should be made to a more specific term. For example, if the gene product being annotated binds to a histone, then ‘histone binding’ [GO:0042393] is the appropriate term.
- Many terms in the Molecular Function ontology implicitly or explicitly imply the binding of a chemical or protein. In these cases, it is unnecessary to co-annotate the binding of the substrates, cofactors or products, as the enzymatic activity is defined by the compounds being bound, if only in a transition state. For example, while annotating to terms like ‘ATPase activity’ [GO:0016887] it is implicit that the gene product binds to ATP and thus it is not necessary to annotate to both ‘ATPase activity’ and ‘ATP binding’ [GO:0005524].

## Biological process

Biological Process describes biological goals accomplished by one or more ordered assemblies of molecular functions. A biological process is not equivalent to a pathway. Specifically it does not represent any of the dynamics or dependencies that would be required to describe a pathway. Examples of broad Biological Process terms include ‘metabolic process’, ‘signaling’ and ‘death’. High-level processes such as ‘cell death’ [GO:0008219] can have both subtypes, such as ‘apoptotic process’ [GO:0006915], and subprocesses, such as ‘apoptotic chromosome condensation’ [GO:0030263] (see Table 1). Experiments describing the phenotypes of mutant genes, genetic interactions and some *in vitro* assays, can all be informative about the biological processes in which a gene product participates (Figure 2).

- On occasion when authors present experimental results for a gene product’s role in a specific type of process, they then extrapolate to infer its role in other related processes. The annotations made from a given article should only be for the processes experimentally demonstrated in that study. For example, if the results show that a gene product can transport serine and threonine, but the authors extrapolate that the gene product can thus transport any amino acid, the gene product should be annotated only to ‘serine transport’ [GO:0032329] and ‘threonine transport’ [GO:0015826] and not to ‘alanine transport’ [GO:0032328], etc.
- Similar to the above example, if the results show a response to a variety of stress conditions, it is best to capture that data with the specific terms rather than annotating to a higher-level term. For example, the *Saccharomyces cerevisiae* gene *HSP12* is annotated to specific terms ‘cellular response to heat’ [GO:0034605], ‘cellular response to osmotic stress’ [GO:0071470] and ‘cellular response to oxidative stress’ [GO:0034599] (22) rather than the high level ‘cellular response to stress’ [GO:0033554]. Grouping terms such as ‘cellular response to stress’ are discouraged from use in direct annotations because an experiment would typically not describe the response to a global stress, but would rather test the response to a specific type of stress.
- Direct versus indirect effect. Many GO Biological Process annotations are assertions based upon mutant phenotypes. When annotating based upon mutant phenotype results, it can be difficult to discern if a gene product is directly involved in the process for which the authors screened (assayed) or if its absence instead results in an indirect or downstream effect. For example if any of the *S. cerevisiae* proteins involved in ‘RNA splicing’ [GO:0008380] are mutated, translation is affected. This is a downstream effect because most of the genes encoding ribosomal proteins have introns (example, yeast ribosomal genes *RPL2A*, *RPL2B*, *RPS11A*, *RPS11B*) and if splicing genes are mutated, these ribosomal genes are not processed thereby affecting ribosomal assembly and hence translation. In this case the genes involved in splicing shouldn’t be annotated to ‘translation’ [GO:0006412]. Determining if a mutant phenotype reflects a direct or indirect effect requires general understanding of the gene products as well as the biological process under investigation. However, in cases where little is known about the gene product or process, or what is known is not easily reconciled with a mutant phenotype, it is the responsibility of the biocurator to accurately reflect the conclusions made from the available experiments. Such annotations should be revisited when new literature becomes available and should be replaced with a more specific term(s) if possible.
- Annotating from gene or protein expression studies. There are many expression studies that measure the levels of RNA molecular species or protein levels when an organism or cell line is exposed to various stimuli. Conclusions from these experiments can suggest that the over-expressed genes or proteins are involved in ‘responding to that stimulus’. However, overexpression does not necessarily imply that those genes or proteins are directly involved in the ‘response to the stimulus’ [GO:0050896]. The ‘response to’ GO terms are intended to annotate gene products that are required for the response to occur and are a direct result of the organism’s reaction to the stimuli (e.g. production of a gene product used to degrade a toxin or signaling to initiate immune cell division in response to a parasite). If nothing else is known about the gene product, it is acceptable to annotate to a child of ‘response to stimulus’ using the IEP evidence code. If more is known about the regulation of the gene product, then that should be taken into account to make a decision about annotating to the ‘response to’ term. It is acceptable to not annotate from such expression studies since changes in expression of a gene product does not in itself indicate its contribution to the function or process. Also, expression studies can seldom support annotations to a Cellular Component or Molecular Function term. Thus IEP should be used to annotate to terms in Biological Process only.
- Annotating to regulation terms in Biological Process. Regulation of a biological process is defined as a role that modulates the frequency, rate or extent of that process.
  ○ To decide if the gene product participates directly in a process or regulates that process, the nature of the process should be studied carefully (Is there a defined pathway? Is it a biochemical pathway and have the gene products that perform the individual steps been identified? Does the gene product being annotated function within the pathway or outside of the pathway to start or stop or change the rate of the process?)
  ○ If it cannot be determined whether the gene product is involved in the process itself or instead in regulation of the process (this can happen if the process is not well defined), then biocurators should annotate to the parent process term. For example, if a mutant phenotype shows that a specific process is missing in an organism but the nature of the function of the gene product is unknown, an annotation should be made to the parent process term. Note that processes in GO are defined to reflect the predominant community view with respect to what is included in the process and what is influencing or regulating the process externally.
  ○ Some gene products can be annotated to both a process and regulation of that process as in the case of positive and negative feedback loops.

## Cellular component

Cellular Component describes locations, at the levels of subcellular structures and macromolecular complexes. Experiments informing Cellular Component annotations include fluorescence microscopy and co-fractionation of complex members. Examples of cellular components include ‘nuclear inner membrane’ [GO:0005637], with the synonym ‘inner envelope’, and the ‘ubiquitin ligase complex’ [GO:0000151] (see Table 1), with several subtypes of these complexes represented.

- Care must be taken when interpreting a subcellular location, as certain tagged proteins may be mistargeted. For example, in Huh *et al.* (23), (see their Supplementary Table S2), the authors list several yeast proteins that were mislocalized to the vacuole or other components upon addition of a molecular tag.
- When a macromolecular complex has been characterized, all subunits of the complex should be annotated to an appropriate complex term in the Cellular Component ontology (example, ‘spliceosomal complex’ [GO:005681] or ‘nucleosome’ [GO:0000786]). Depending on the nature of the experiment, annotation to a complex can either be made using the IDA evidence code or the IPI evidence code. For example, if an author purifies a complex and then investigates the constituent gene products, a curator would use the IDA evidence code for annotation. If the authors instead perform protein-binding assays to show that a gene product binds to other members of the complex, then the IPI evidence code should be used with appropriate targets included in the *WITH/FROM* column (see below).
- There are several terms in the Cellular Component ontology in the format ‘x part’ (e.g. ‘nuclear part’ [GO:0044428]; ‘membrane part’ [GO:0044425] etc.). These terms were added to make the ontology *is_a* complete (i.e. ontologically correct). Without additional qualifiers, annotation to these terms conveys no more information than annotation to the parent terms. Hence, these terms should not be used in making manual annotations.

## Additional information about the GO term (annotation extensions)

Often, an article will contain more detailed information than existing GO terms can fully represent. In many such cases, biocurators may request new more specific terms to be added to the ontology, but new GO terms may not always be the preferred solution. Rather, some information, such as the substrates of a protein kinase or the cell type in which a gene product has a particular localization, is best-captured using annotation extensions (also referred to as ‘column 16’ after its position in the GAF, Table 3). Additional information captured in this column provides more biological context to the GO annotation.

An annotation extension has two parts: an entity identifier for the object that is used to increase the specificity of the annotation (e.g. identifiers for a gene, gene product, GO term or a term from an external ontology such as a cell type or anatomy ontology), and a relation that connects the ‘primary’ GO term to the entity represented by the identifier. The information captured in GO annotation extensions encompasses several types of effector–target relationships.

- The substrates of a function such as the target of a protein kinase. For example, the *S. pombe* win1 (SPAC1006.09) protein has been annotated to ‘MAP kinase kinase kinase activity’ [GO:0004709] with the extension ‘*has_direct_input* (pombase:wis1)', where the *S. pombe* protein wis1 is the substrate of win1.
- Activators and inhibitors, using the relationships *activated_by* and *inhibited_by*.
- Regulation targets of signaling pathways or transcription factors. For example, the *S. pombe* gene map1 is annotated to ‘positive regulation of mating-type specific transcription from RNA polymerase II promoter’ [GO:0001197] with the extension ‘*has_regulation_target* (PomBase:SPMTR.02)' indicating that SPMTR.02/matPi is the target of the regulation event.
- Spatial aspects of processes or localizations, as in a specific cell or tissue type as represented in the Cell Type Ontology (24), e.g. *occurs_in* [CL:0000182], where CL:0000182 identifies the cell type ‘hepatocyte'.
- Temporal aspects of a process or developmental stage, e.g. ‘*happens_during*' for mitosis. For example, the *S. pombe* gene mug27 is annotated to ‘septation initiation signaling cascade’ [GO:0031028] with the extension ‘*happens_during* meiotic cell cycle’ [GO:0051321] implying that mug27 is involved in septation initiation signaling cascade that happens during meiotic cell cycle.

An annotation may have one or more extensions, using the same or different relations. It is thus possible to capture multiple substrates of a kinase, for example. Compound extensions are also allowed, making it possible to indicate that two or more extensions apply simultaneously. For example, a gene product that is involved in a process only when it localizes to the nucleus, and only during S-phase of the cell cycle, can be annotated to a process term plus the extension ‘*occurs_in* nucleus’, ‘*during* S phase of mitotic cell cycle’. A list of allowed relationships are available in the go_annotation_extension_relations.obo file (http://viewvc.geneontology.org/viewvc/GO-SVN/trunk/ontology/extensions/go_annotation_extension_relations.obo) while the format for the various database identifiers can be found in the GO cross reference file (http://www.geneontology.org/doc/GO.xrf_abbs) (manuscript in preparation).

## Choice of evidence code

Four different categories of evidence codes are available for manual curation: experimental, computational analysis, author statements and curatorial statements (details in Table 2, Figure 2).

- Use of an experimental evidence code indicates that the cited article reported results that support the association of a GO term from characterization of a gene or gene product.
- Evidence codes in the computational analysis category imply that the annotation was inferred based on *in silico* analysis of the gene or gene product sequence and/or other data as cited in the reference. Biocurators can also perform *in silico* analysis, independent of a published article, to infer an annotation, in which case a GO Reference (GO_REF) that describes the methods used by the biocurator is used as reference.
- Author statements include assertions made anywhere in the cited article, including the Introduction and Discussion. These evidence codes were made available by the GOC because during the initial stages of the project; curation of such statements was an easy way to get a good volume of annotations quickly. However, annotations using these evidence codes are now being replaced by those citing direct evidence. Use of author statement codes is discouraged and so they are not described in detail here.
- Curatorial statements indicate that the biocurator reviewed the information and made the appropriate annotation decision. IC and ND are curatorial statement codes. The ND evidence code, which has been described earlier in the article, is used to indicate that there is no biological data available to infer any GO term for that gene product. The IC evidence code can be used in two different scenarios. The first case includes those instances where an annotation is not supported by any direct evidence, but can be reasonably inferred by a biocurator from other GO annotations, for which evidence *is* available. For example, if a gene product is shown experimentally to have the function of ‘sequence-specific DNA binding RNA polymerase II transcription factor activity’ (GO:0000981), and there is no direct evidence for the cellular location of the gene product, then it is within general knowledge that this function takes place in the nucleus and thus the biocurator can infer the gene product’s location. Both annotations will use the same published article as reference and in addition the IC annotation will include the GOID used by the biocurator for the inference in the *FROM* (column 8 in the GAF 2.0 file, Table 3). In the second case, a curator infers an annotation based on evidence from multiple sources of evidence/GO annotation as described below.

## Data supporting the evidence code

In addition to the evidence code that reflects the type of experiment leading to an annotation, the GOC provides two ways to capture additional evidence information for an annotation: the qualifier and the *WITH/FROM* column. A qualifier can be used to augment the interpretation of the GO term. Three qualifiers are available: *colocalizes_with*, *contributes_to* and *NOT*. These are found in the *QUALIFIER* column of the GAF 2.0 format (Table 3).

## QUALIFIER

- Sometimes, gene products are transiently or peripherally associated with an organelle or complex. These results can be annotated to the relevant Cellular Component term along with the *colocalizes_with* qualifier. The *colocalizes_with* qualifier can be used only with the Cellular Component ontology. For example, the *S. pombe* protein clp1 is a nucleolar protein but transiently associates itself with the ‘actomyosin contractile ring’ [GO:0005826] (25). Hence clp1 is annotated to this term with the *colocalizes_with* qualifier.
- The *contributes_to* qualifier can be used only with Molecular Function terms. Sometimes complexes are shown to have an activity, but the activity of each subunit is not shown. In such cases, individual subunits that are part of a complex can be annotated to terms that describe the function of the complex. If the activity of the complex is associated with a single subunit and the other subunits serve either as regulatory subunits or to keep the complex together, then the subunits should be annotated to those specific activities. *Contributes_to* is not needed to annotate a catalytic subunit. Furthermore, *contributes_to* may be used for any noncatalytic subunit, whether the subunit is essential for the activity of the complex or not. In another usage, if two or more subunits of a complex are required for the catalytic activity of the complex, then all those subunits get annotated to the corresponding Molecular Function term with the *contributes_to* qualifier. The gene products annotated to function terms with the *contributes_to* qualifier should also be annotated to the complex term in the Cellular Component that has that molecular function. For example, the subunits of the *S. cerevisiae* mitochondrial respiratory chain complex III are all annotated to the Molecular Function term ‘ubiquinol-cytochrome-c reductase activity’ [GO:0008121] with the *contributes_to* qualifier (26) and to the complex term ‘mitochondrial respiratory chain complex III’ [GO:0005750] in the Cellular Component ontology. This qualifier is not used with terms in Biological Process ontology because biological processes are a collection of molecular events and by default gene products contribute to the whole process.
- The negative of a GO term, the *NOT* qualifier. This qualifier is used to explicitly denote that the gene product is *not* associated with the function, process or component represented by the GO term. This qualifier is used when a gene product is expected to have a function, but has been shown experimentally *not* to have the enzymatic activity; in this case the gene product can be annotated as *NOT*. For example, the NOT qualifier is used to indicate that the *Caenorhabditis elegans* gene C42C1.11a.2 was experimentally shown to NOT have ‘leukotriene-A4 hydrolase activity’ [GO:0004463] despite strong homology to the human leukotriene A4 hydrolase (27). Annotations that use the NOT qualifier can be particularly informative for evolutionary studies that wish to explore the gain and/or loss of gene product activity.

## WITH/FROM column

- The *WITH* column is required for *Inferred from Electronic Annotation* (IEA), IGI, IPI, ISS, *Inferred from Sequence Alignment* (ISA) and *Inferred from Sequence Orthology* (ISO) codes (Table 2).
- For example, when using ISS, the *WITH* column should be used to indicate the identifier of the gene product used for the sequence or structural comparison. For annotations based on sequence comparisons, it is important to confirm that the protein used for the sequence comparison was experimentally verified to have that function and has a GO annotation reflecting that experimental finding. If a GO annotation is missing please report this to the GO consortium (go-helpdesk@geneontology.org).
- Likewise, for IPI and IGI codes, the *WITH* column should be used to indicate the interacting gene product or gene respectively. Multiple identifiers can be entered in this field.
- The *FROM* value is used to provide supporting information for the IC evidence code. For example if a Molecular Function annotation is made to ‘sequence-specific DNA binding RNA polymerase II transcription factor activity’ [GO:0000981] with experimental evidence, and a biocurator deduces that the gene product thus resides in the nucleus, then the component annotation to nucleus is made with the *FROM* value GO:0000981.
- In many cases a GO term can be inferred from just one other annotation, but occasionally a curator can also infer an annotation to a term based on evidence from multiple sources of evidence/GO annotation. The *FROM* value in these annotations will therefore supply more than one GO identifier, obtained from the set of supporting GO annotations assigned to the same gene/gene product identifier which cite publicly available references and the annotation would have an unpublished GO reference (GO_REF:000036) in its Reference field.

## Suggested reading

For examples of how GO annotations have been developed and how these guidelines have been put into practice please consult the following articles. The work on biofilm and filamentous growth in *Candida* (28), heart development (29), a case study of focused curation for renal and cardiovascular research (30) and in depth curation of the peroxisome proteome in humans (31) will be instructive for learning about curation of the literature to create GO annotations.

## Conclusions

The goal of the GOC is the unification of biology by creating a nomenclature used for describing the functional characteristics of any gene product, protein or RNA, from any organism. The GOC provides the research community a comprehensive resource of functional information on gene products. Toward this end, the GOC provides ontologies, guidelines to make the gene product-to-GO term associations and standardized formats to publish these annotations. This guide describes the methods used to create one of the two types of annotations that can be made with GO terms: manual curation. Consistency of GO annotations is paramount to ensure the quality of any analysis using the annotations. An understanding of the requirements and strategies associated with the three aspects of the GO with those of the different evidence codes can ensure manual annotations will be an accurate representation of the published results. Our hope is that these guidelines will provide encouragement and assistance to researchers to annotate their favorite gene products, enriching both the quality and quantity of GO annotations available via the GOC.
